# Mercury and selenium in developing and mature fruiting bodies of *Amanita muscaria*

**DOI:** 10.1007/s11356-021-14740-6

**Published:** 2021-06-21

**Authors:** Anetta Hanć, Alwyn R. Fernandes, Jerzy Falandysz, Ji Zhang

**Affiliations:** 1 Department of Trace Element Analysis by Spectroscopy Method, Adam Mickiewicz University, Umultowska 89b, 61-614 Poznań, PL Poland; 2 School of Environmental Sciences, University of East Anglia, Norwich, NR4 7TJ UK; 3 Environmental Chemistry and Ecotoxicology, University of Gdańsk, Gdańsk, Poland; 4 Environmental and Computational Chemistry Group, School of Pharmaceutical Sciences, University of Cartagena, Zaragocilla Campus, 130015 Cartagena, Colombia; 5 Medicinal Plants Research Institute, Yunnan Academy of Agricultural Sciences, Kunming, 650200 China

**Keywords:** Fly agaric, Ectomycorrhizal, Mushrooms, Methylmercury, Seleno-compounds, Sporocarp development

## Abstract

Both mercury (Hg) and selenium (Se) occur in many mushroom species, but the morphological distribution of these elements during different developmental stages of the fruiting bodies is not known. Although *Amanita muscaria* can be consumed after suitable processing, they are often ignored by mushroom foragers, leaving an abundance for investigative study. Multiple specimens in each of six developmental stages (button to fully mature) were collected in excellent condition during a single morning from the same forested location and composited. With an average of 30 specimens per composite, and low temporal, spatial, and measurement uncertainty, the data are likely to be representative of the typical concentrations of Hg and Se for each developmental stage. Hg (range 0.58–0.74 mg kg^-1^ dry weight cap; 0.33 to 0.44 mg kg^-1^ dw stipe) and Se (range 8.3–11 mg kg^-1^ dw cap; 2.2 to 4.3 mg kg^-1^ dw stipe) levels were observed to vary during the developmental stages, and the variability may relate to the demands in growth. In common with some other species, the lower stipe concentrations may be consistent with nutrient/contant transport and support functions. Both Hg and Se levels were lowest during periods of maximum sporocarp growth. Selenium occurs at almost an order of magnitude greater levels than Hg. Due to its role in mitigating the effects of Hg toxicity, this property is of significance to those who consume the species either for nutritional, medicinal, or recreational purposes, although the losses of both these elements during processing are not known.

## Introduction

*Amanita muscaria* (L.) Lam*.* is an ectomycorrhizal fungus, popularly and better known worldwide as the fly agaric mushroom. It was notorious in earlier decades for its hallucinogenic properties—all parts of the mushroom have psychoactive properties, caused by some of its related constituents, ibotenic acid, muscarine, muscazone, and muscimol (an unsaturated cyclic hydroxamic acid) which may be more abundant in the flesh just below the cuticle. Historically used in shamanistic rituals in some northern European and Asian cultures, *A. muscaria* has also been reportedly (Guzmán 2008) used in traditional Mexican medicine as a diuretic and purgative and for the treatment of epilepsy. The mushroom is less well known as a food that is foraged or collected for recreational purposes. Detoxification is achieved by boiling of the flesh (a couple of times) with rejection of the water (Rubel and Arora 2008). It is consumed in parts of Europe, North America, and Asia, particularly Japan, where it may be consumed after salting and pickling (Phipps et al. 2000).

*Amanita muscaria* is native to coniferous and deciduous forests across the temperate Northern Hemisphere but is also found in other regions in the Southern Hemisphere (Hawkeswood 2006) where it was inadvertently introduced through pine and other plantations. It exists in biochemical symbiosis with the roots of conifers including pine, spruce, fir, birch, and cedar, but the mycorrhizal associations may be tripartite, involving other fungal species as well. It was reported (Yun and Hall 2004) that rhizomorphs of *Boletus edulis* Bull. were often observed closely intertwined with *Amanita*, forming composite mycorrhizas which may facilitate the flow of nutrients between the two species.

Mushrooms, in general, are known to bio-concentrate a whole range of metals and metalloid elements, some of which have sometimes been used as indicators of local pollution as they reflect pollutant distributions in the substrate. In ectomycorrhizal species such as *Amanita,* metallic and metalloid elements may be actively or passively absorbed by the mycelium from the surrounding soil or substrate. The build-up of heavy metals is undesirable but not all elemental accumulations are harmful, and some have essential physiological functions in the flesh of mushrooms. Potassium, for example, functions as an intracellular cation for osmotic regulation of the water content and as a co-factor in enzymes. As part of the biochemical symbiosis in *Amanita*, the absorbed trace elements are used by the mycelium but may also be shared with the ectomycorrhizal symbiont(s). In return the fungus obtains access to photosynthesis products, e.g., simple carbohydrates. Forest ecosystems have a high diversity of mycorrhizal associations and are subject to complex multi-species food chain networks (Luo et al. 2014; Van der Heijden et al. 2015).

This characteristic of sourcing nutrition from the surrounding soils or substrate bears an unfortunate side effects; it makes mushrooms vulnerable to contaminant uptake. In common with many species of fungi that grow in habitats that are contaminated with mercury, either of geogenic or anthropogenic (mining or other industrial) origins, *A. muscaria* efficiently bio-concentrates Hg. Reported bioconcentration factors (BCFs) were in the range of 8.3 to 51 in the caps and from 4.1 to 28 in the stipes (Falandysz et al. 2007; Lipka et al. 2018), but several other *Amanita* spp. in Canada showed lower BFCs of 3.1 (caps) and 1.7 (stipes) (Nasr and Arp 2011). Fischer et al. (1995) noticed a higher potential of *A. muscaria*, collected from a former cinnabar mining site, to bio-concentrate methyl mercury (MeHg) (BCFs 3.0–10) relative to inorganic Hg (BCF was 0.48–1.0), which was associated to the humus (organic) content of the soil layer.

Both inorganic mercury and MeHg occur in foods in varying amounts, with relatively high levels in foods of marine and animal origin (EFSA 2012). Inorganic Hg is highest in terrestrial foods such as wild mushrooms. Some reported Hg dry weight (dw) concentrations (Falandysz et al. 2015; Melgar et al. 2009; Seeger 1976), for example, of the popular genus *Boletus* were:

- Up to 22 mg kg^-1^ dw in the caps and 8.4 mg kg^-1^ dw in stipes of pooled fruiting bodies of *B. bainiugan*.
- 5.7 (1.0–17 mg kg^-1^ dw; n = 8) in whole *B. edulis* with caps showing 1.1 ± 1.4 to 7.6 ± 3.1 (0.02–14 mg kg^-1 ^dw) and stipes from 0.82 ± 0.71 to 3.8 ± 1.8 (0.03–6.7 mg kg^-1^ dw; n = 173).
- *B. pinophilus* concentrations were 4.5 ± 5.0 mg kg^-1^ dw (in hymenophore 6.9 ± 8.3 mg kg^-1^; dw  n = 13).
- *B. reticulatus* showed up to 4.4 ± 1.3 mg kg^-1^ dw in caps and up to 2.5 ± 0.9 mg kg^-1^ dw in stipes (n = 15).

Although much of the terrestrial Hg in mushroom habitats is inorganic, it is well-known that it can be transformed by microorganisms such as anaerobic bacteria that facilitate methylation to MeHg. Both forms are assimilated by fungal fruiting bodies with MeHg occurring at up to 15% of the total Hg (THg) content in some species, according to some sources (Kalač and Svoboda 2000).

Other elements also occur in mushrooms as previously mentioned, and in the context of Hg and MeHg toxicity, the co-occurrence of selenium (Se) is of particular interest, because of the long-reported “protective” effect of its biochemical forms, against MeHg toxicity. In effect, MeHg which is normally found in kinetically labile bonds to thiols in cerebral tissues is promiscuously capable of scavenging Se from selenoproteins that are functional in reversing and preventing oxidative damage in these tissues (Ralston and Raymond 2018). This targeted binding compromises the biological functions performed by selenium compounds, but the resulting depletion of Se compounds in the brain can be balanced by mobilizing reserves from other tissues, providing that the affected subject’s diet can support the required balance with Se-rich foods. Alternatively, supplemental Se could be administered to counter the adverse consequences of high MeHg levels by supporting essential brain selenoenzyme activities. Clearly, it would be beneficial to know the Hg-Se balance in mushroom species that are consumed, either as medicinal products or perhaps more particularly for species that are foraged in the wild, either out of necessity or for recreational purposes. It may even inform risk management strategies in areas with elevated mercury backgrounds or in regions that show Se deficiencies in the diet (Wąsowicz et al. 2003).

The amount of published data on the mineral contents of fungi continues to increase, but little is known about how the distribution varies within the morphological parts, or how this distribution changes during the developmental stages; indeed some of the scarce available information is contradictory (Hinneri 1975; Falandysz et al. 2019 and Falandysz et al. 2020; Melgar et al. 2009; Thomet et al. 1999; Komárek et al. 2007; Lavola et al. 2011; Nasr et al. 2012).

Mercury occurs in higher concentration in caps than stipes of the fruiting bodies and is largely concentrated in hymenophore—gills and tubes (Falandysz et al. 2007b; Melgar et al. 2009; Kavčič et al. 2019; Seeger et al. 1976). The metallic and metalloid elements such as Ag, As, Cd, Cs, Cu, Fe, Hg, K, Mg, Rb, Se, and Zn are favorably distributed in caps and Cr, Na, Li, Cr, and U in stipes of fruiting bodies, while Ba, Co, Ni, Pb, Sr, Tl, and V shows an equal distribution between morphological parts of *A. muscaria*, but this may be influenced by some site-specific geochemical conditions of the soil substrata which can be mirrored in the BCF values for the sites (Drewnowska et al. 2013; Falandysz et al. 2007a, Falandysz et al. 2020; Lipka et al. 2018; Lipka and Falandysz 2017).

In an attempt to clarify Hg and Se occurrences, this study investigated different developmental stages of the fruiting bodies of *A. muscaria*. This species occurs commonly in the forests of Europe during the late summer and autumn months along with others edibles such as *B. edulis*, *Leccinum* spp., and others including the invasive *Aureoboletus projectellus* (Murrill) Halling (previous name *Boletus projectellus*) occurring in large numbers in recent years in Poland and which are preferred by mushroom foragers and recreational pickers. The resulting abundance of unpicked *A. muscaria* allows expedient collection of specimens in different stages of development.

## Materials and methods

### Sampling

The sampling area was located in the Kolbudy forest District (including Pomlewo village and surroundings) which is part of the rural Przywidz commune in Pomeranian Voivodeship, Poland. The forest contains mixed growths of deciduous and coniferous trees which are a typical habitat of *A. muscaria.* Forest cover was dominated by young, downy birch (*Betula pubescens *Ehrh.) interspersed with Scots pine (*Pinus sylvestris *L.) and Norway spruce (*Picea abies* (L.) H.Karst.).

In order to minimize any temporal effects, all of the specimens of *A. muscaria* were collected in a single morning in late September 2015, within a few hours of each other. There was good availability, and only fruiting bodies in excellent condition were selected. Immediately at the site of collection, the mushrooms were freed from forest floor litter and other debris, using a plastic knife. After collection, the fruiting bodies were separated by visual examination into six groups that reflected their stage of development with class 1 containing the youngest and class 6 containing the most mature specimens. The numbers of specimens within a group varied from 16 to 54 individuals.

**Fig. 1:**
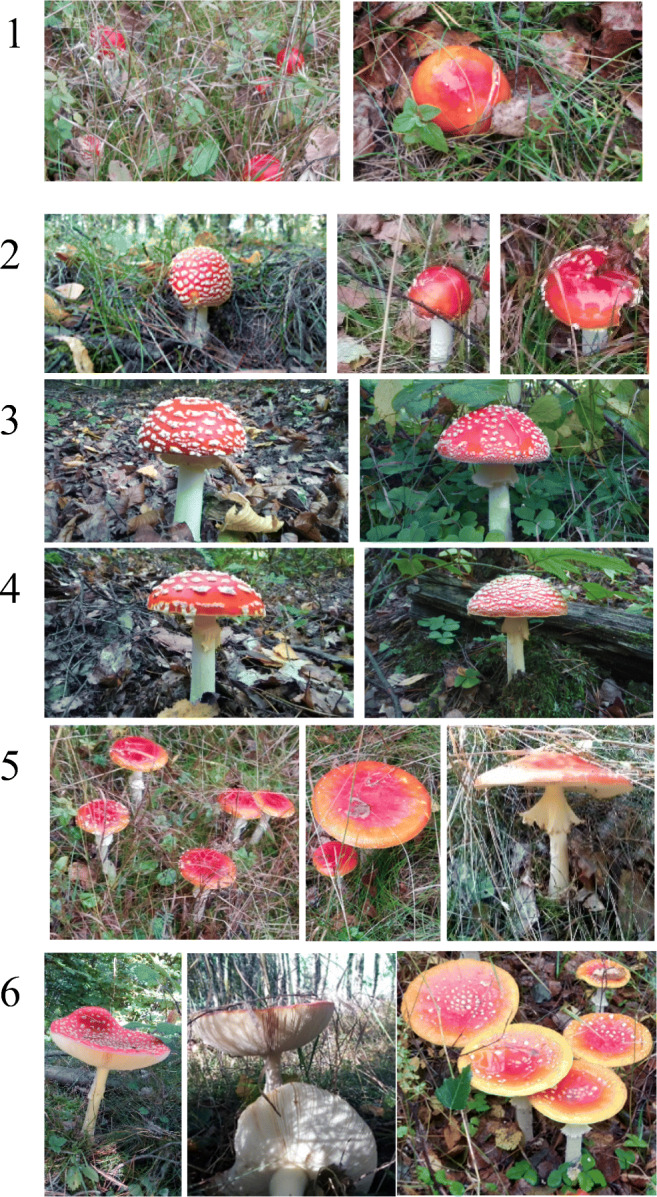
The six developmental stages of *A. muscaria* from button to mature, sampled in this study (color for online publication only)

Class 1 was made up of 54 specimens with the smallest fruiting bodies (button stage).Class 2 contained 27, and class 3, an intermediate stage of development, was the smallest pool with 16 individuals. Class 4 and class 5 contained 30 and 22 individuals, respectively, and class 6 which consisted of fully mature specimens was made up of 32 individuals (Fig. 1). The caps and stipes within each group were separated and pooled accordingly.

### Sample preparation

Individual pooled groups were dried separately to a constant mass at 65 °C. The dried material was individually ground to a very fine powder using a blender with a glass jar and ceramic blades. The blender was cleaned between each group in order to minimize cross contamination. The pulverized samples were deposited in polyethylene bags that were sealed and placed into larger polyethylene bags which were stored in the laboratory’s sample depository under dry and clean conditions at room temperature, until further analysis.

### Analysis and quality control

Analysis of the collected samples was carried out using microwave-assisted acid digestion of dried sample material followed by measurement using cold vapor atomic absorption spectroscopy (CV-AAS) in the case of Hg and inductively coupled plasma mass spectrometry (ICP-MS) for Se. Procedural blanks and a reference material were included in analyses to order to ensure reliability and confidence in the generated data. In the immediate period before analysis, the batch of fungal materials was dried for 12 h at 65 °C using an electrically heated laboratory oven. Around 10–20 mg aliquots of the dried material was taken for direct Hg determination, whilst for Se (and other elements), around 500 mg aliquots was used. These were weighed into PTFE containers and digested in a 5-mL solution of concentrated nitric acid (Suprapure, 65%, Merck, Germany) using a pressure-assisted microwave digestion system, along with three batch procedural blanks. The resulting solutions were transferred to pre-marked acid-clean plastic tubes and diluted to 10 mL with deionized water (18 MΩ-cm) (TKA Smart2Pure, Niederelbert, Germany).

The Hg content was determined using CV-AAS (MA-2000 mercury analyzer—Nippon Instruments Corporation, Takatsuki, Japan) by direct sample thermal decomposition combined with a gold wool trap and amalgamation of Hg vapor and Hg desorption. The element was quantitatively determined at a wavelength of 253.7 nm with instrument operation in both high (25 to 150 ng Hg per sample) and low (3 to 20 ng Hg per sample) modes. Calibration curves were prepared by injecting 1.0 mg mL^-1^ Hg standard solutions of 25, 50, 100, 150, and 200 μL (high mode) and 3, 5, 10, 15, and 20 μL (low mode). The reference materials (Table 1) were prepared and examined in the same way.

**Table 1:** Assigned and determined and results on the trace element concentrations in biological control materials (mg kg^-1^ dw) (n = 3)

Element	Control material*			
	CM CS-M-3		CRM CS-M-4	
	Assigned	Determined	Assigned	Determined
Hg	2.849±0.104	2.759±0.075	0.463 ± 0.024	0.438±0.031^#^
Se	16.21 ± 0.85	17.43 ± 1.36	1.29 ± 0.08	1.29 ± 0.03

Notes: *Control reference materials: CM CS-M-3 (dried mushroom powder *Boletus edulis*) and CM CS-M-4 (dried mushroom powder *Leccinum scabrum*)—produced by the Institute of Nuclear Chemistry and Technology, Warsaw, Poland; WD (without data); ^#^(n = 11)

Selenium was determined using ICP-MS, on an ELAN DRC II ICP-MS inductively coupled plasma mass spectrometer (PerkinElmer, SCIEX, Canada). The instrument was equipped with a Meinhard concentric nebulizer, cyclonic spray chamber, dynamic reaction cell, Pt cones, and a quadruple mass analyzer and operated using the dynamic reaction cell (DRC). The DRC consists of an enclosed quadrupole analyzer that is located between the ion lens system and the main analyzing quadrupole. The use of this technology reduces most polyatomic interferences to near background levels, which provides a significant improvement in detection limits. The instrument was operated in multi-elemental mode, and in addition to Se, a number of other elements were also determined. Calibration curves using standard solutions (0.1 μg L^-1^ to 100 μg L^-1^) were constructed in a similar manner to the Hg determination.

The fungal materials were analyzed in duplicate (Se) and triplicate (Hg) for confirmation of concentration. The analytical methodology has been fully validated and reported earlier (Falandysz et al. 2015 and Falandysz et al. 2020) after peer review. The quality of measurement was continually assessed and controlled during the period of analysis using freshly prepared standard solutions, routine instrument calibration, and simultaneous analysis of the batch procedural blanks and certified materials with each analytical cycle.

## Results and discussion

The concentrations of Hg and Se in the caps and stipes of composited samples of *A. muscaria* at the six stages of growth of the sporocarp are given in Table 2. The data are also expressed as molar (μM kg^-1^dw) concentrations.

**Table 2:** Distribution of Hg and Se in dehydrated *A. muscaria* over six stages of sporocarp development

Stage	Part	n	Hg (mg kg^-1 ^dw)	Se (mg kg^-1 ^dw)	Hg (μM kg^-1 ^dw)	Se (μM kg^-1^ dw)
S-1	Cap S-1	54	0.74 ± 0.00	11 ± 0.0	3.69	139
S-2	Cap S-2	27	0.66 ± 0.01	9.1 ± 0.4	3.29	115
S-3	Cap S-3	16	0.67 ± 0.01	8.3 ± 0.2	3.34	105
S-4	Cap S-4	30	0.58 ± 0.00	8.6 ± 0.2	2.89	109
S-5	Cap S-5	22	0.62 ± 0.01	11 ± 0.0	3.09	139
S-6	Cap S-6	32	0.74 ± 0.04	9.8 ± 0.3	3.69	124
	Mean, Cap		0.67	9.63	3.33	122
S-1	Stipe S-1	54	0.44 ± 0.01	4.3 ± 0.2	2.19	54
S-2	Stipe S-2	27	0.34 ± 0.01	2.8 ± 0.1	1.69	35
S-3	Stipe S-3	16	0.33 ± 0.01	2.2 ± 0.2	1.65	28
S-4	Stipe S-4	30	0.34 ± 0.01	2.5 ± 0.1	1.69	32
S-5	Stipe S-5	22	0.36 ± 0.01	4.1 ± 0.2	1.79	52
S-6	Stipe S-6	32	0.38 ± 0.01	3.4 ± 0.2	1.89	43
	Mean, Stipe		0.37	3.22	1.82	41

### Mercury occurrence in *A. muscaria*

**Fig. 2:**
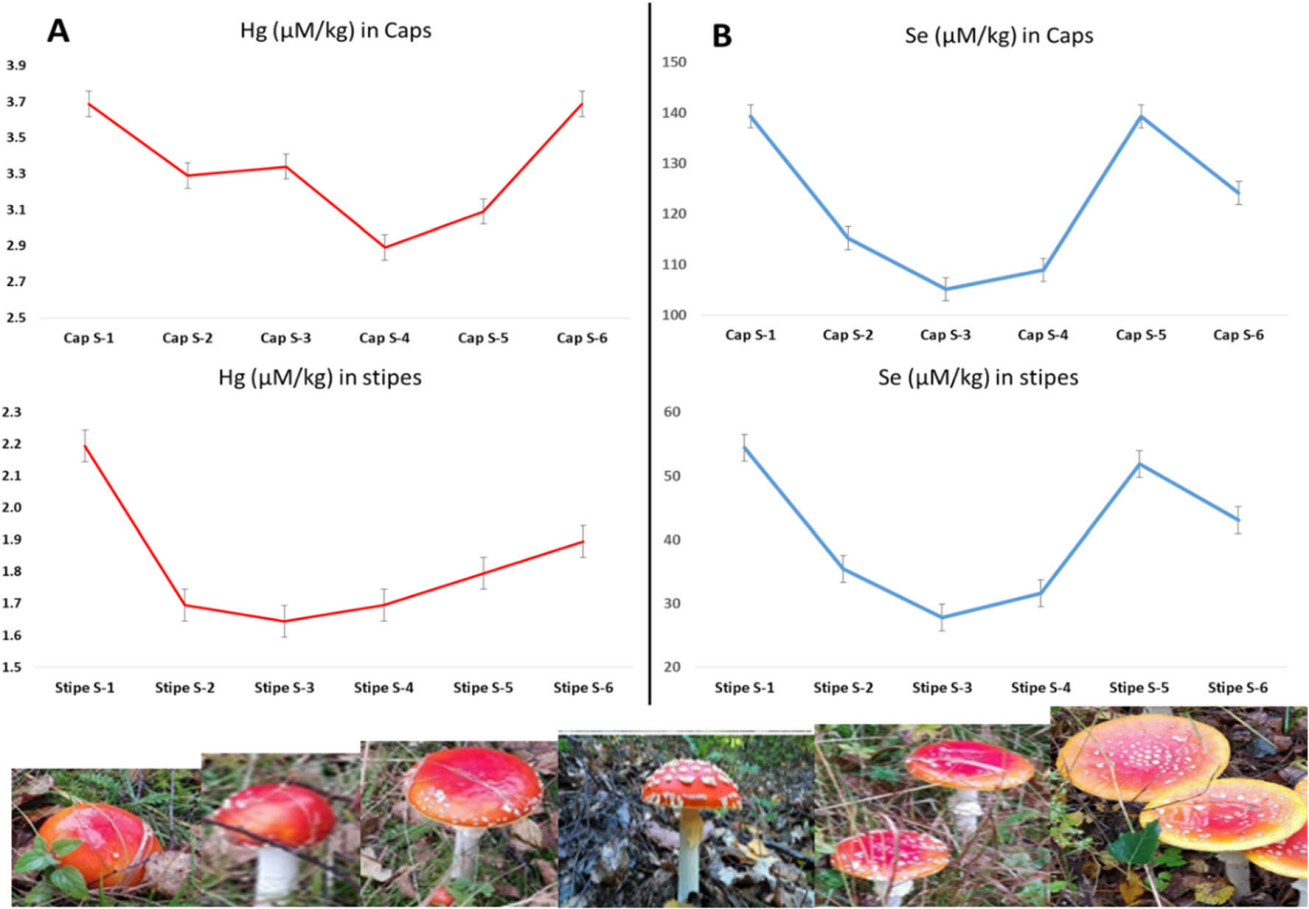
Molar concentrations of Hg (**A**) and Se (**B**) in caps and stipes of *A. muscaria* at different developmental stages (color for online publication only)

Examination of Table 2 shows that the Hg concentrations in *A. muscaria* ranged from 0.58 to 0.74 mg kg^-1^ dw (equivalent to 2.89 to 3.69 μmol kg^-1^ dw) in the caps depending on the maturity stage of the fruiting body. The corresponding range for the stipes was 0.33 to 0.44 mg kg^-1^ dw (1.65 to 2.19 μmol kg^-1^ dw). This initial view of the data suggests that mercury uptake may be stable with continuous absorption by mycelia and translocation at a similar rate to stems and caps of the fruiting bodies. However, closer examination of the data by plotting Hg concentrations against the development stage (Fig. 2) shows similar inflections in the curves for both caps and stipes, with concentrations decreasing from the button stage to mid-growth stages before recovering again in the mature stage 6. It is worth noting that each data point represents up to many tens of samples, with a low variability of measurement (the uncertainty is generally ± 0.01 mg kg^-1^ dw). The other potential source of variability in the metal content of the fruiting bodies—the soil and substrate was also minimized in this study by collecting all specimens from the same rural location without active sources of pollution, at the same time. Thus, the concentrations measured for each development stage are likely to be reliable estimates of the elemental contents. The differences observed suggest that although mycelial uptake of mercury remains constant, the weight gain during the early stages of rapid growth of the sporocarp may dilute the Hg concentrations in intermediate, immature specimens, before the concentration recovers in the mature stage. The lower concentrations in the stipes have been reported in other studies (Drewnowska et al. 2013) and occur at all development stages with a cap to stipe concentration quotient (Q_C/S_) of 1.8 which is the same as that reported earlier by Drewnowska et al. (2013). This is consistent with the functionality of the stipes which consist of sterile hyphal tissue that provides structural support and also serves to transport nutrients to the cap.

### Changes in selenium concentration during sporocarp development

As an essential micronutrient, Se concentrations in the same composite samples of *A. muscaria* were approximately an order of magnitude greater than Hg, and depending on the stage of development, ranged from 8.3 to 11.0 mg kg^-1^ dw (equivalent to 105 to 139 μmol kg^-1^ dw) in the caps and 2.2 to 4.3 mg kg^-1^ dw (equivalent to 28 to 54 μmol kg^-1^ dw) in the stipes. Analogous plotting of the data (Fig. 2B) reveals very identical curve shapes for both the caps and the stipes. These shapes suggest a similar depletion of Se in caps and stipes during the first stages of growth before recovery to initial button stage concentrations by stage 5. However, unlike the curves for Hg, the Se concentrations drop again, by similar proportions in both caps and stipes (11% in the caps and 17% in the stipes). Unlike mercury which is not known to serve any function in mushrooms, Se is functional and exists as several organic selenium-containing amino acids such as selenocysteine, selenomethionine, Se-methylselenocysteine in selenium-dependent enzymes (selenoenzymes/selenoproteins), selenite and several other unidentified seleno-compounds in widely varying proportions depending on the mushrooms species (Falandysz 2008, 2013). The amino acids such as selenocysteine are reported to serve known or predicted oxidoreductase functions (Mariotti et al. 2018). It is not currently known whether the maturing and dispersal of spores during the final stage makes a greater demand on seleno-compounds corresponding to the reduction observed in the mature (stage 6) group. Given the similarity of the plotted curves for Se in the caps and stipes, it is unsurprising that there is good correlation of Se content at different development stages, with a correlation coefficient of 0.996.

### Comparative profile

There are only a handful of other studies that have investigated Hg and or Se in *A. muscaria* including samples from Poland (Table 3) (Falandysz et al. 2007a, b; Stijve 1977; Watkinson 1964). In the first study of Se in *A. muscaria*, two specimens from New Zealand showed Se in concentrations of 16.8 and 17.8 mg kg^-1^ dw (Watkinson 1964). In a study published in 1977 by Stijve (1977), specimens collected near Lake Geneva in Switzerland showed a Se concentration of 2.90 (mg kg^-1^ dw (range 1.37–4.84; n = 3). The Se distribution between fleshy parts in pools of 15 did not differ between caps (3.96 mg kg^-1^ dw) and lamellae (4.04 mg kg^-1^ dw) but was lower in the stipes at 1.23 mg kg^-1^ dw (Stijve 1977). The concentration of Hg in the caps ranged from 0.17 mg/kg for a composite sample collected from the Bydgoszcz area of North Central Poland in 2000 to 0.79 mg kg^-1^ dw for a similar composite harvested in the Masuria region. The corresponding range for the stipes was 0.18 to 0.51 mg kg^-1^ dw also for the Masuria region. The average data from the current study are similar to those for the sample collected from the Masuria region in 1999. The current study showed the highest cap concentrations for Se compared to the literature data, which ranged from 4.1 to 9.8 mg kg^-1^ dw (stipe concentration range—1.4 to 4.7 mg kg^-1^ dw). The data from these studies has been summarized in Table 3, although not all studies included separate measurements for caps and stipes.

**Table 3:** Summary of Hg and Se concentrations (mg kg^-1^ dw) from literature studies on *A. muscaria*.

Location	No. samples	Part	Hg (mg kg^-1^ dw)	Se (mg kg^-1 ^dw)	
					Reference
This study, Pomerania, Poland	32	Cap	0.74	9.8	-
		Stipe	0.38	3.4	-
New Zealand	2	Whole	-	16.8 - 17.8	Watkinson 1964
Near Lake Geneva, Switzerland	15	Cap	-	3.96	Stijve 1977
		Stipe	-	1.23	
Morąg, Poland	15	Cap	0.35	4.6	Falandysz et al. 2007
		Stipe	0.18	1.4	
Giżycko, Poland	15	Cap	0.32	5	
Bydgoszcz, Poland	14	Cap	0.17	4.1	
Masuria, Poland	15	Stipes	0.35	4.7	
Pomerania, Poland		Stipes	0.25	4.4	
Masuria, Poland 1999	15	Cap	0.79	-	Drewnowska et al. 2013
		Stipe	0.51	-	

The uptake of non-essential elements such as mercury in mushrooms is in many ways dependent on the abundance of these elements in the substrate in which they grow, which is in turn dependent on the proximity to sources. Other dependent variables are the species of mushroom and the age of the fruiting bodies and mycelia. However, some species (e.g., *Clitocybe, Agaricus, Lepista, Boletus*) show a tendency to bio-accumulate elements like Hg even if the substrate is not particularly rich in this element (Falandysz et al. 2002). The uptake of other chalcophile elements (surficial elements that associate with sulfur) in *A. muscaria* at different stages of development was recently described (Falandysz et al. 2020). The data indicates variability with uptake depending on the element. The concentrations of some elements such as cadmium (Cd) and silver (Ag) remain broadly unchanged over the different stages of development, but the concentration of others such as lead (Pb) and antimony (Sb) halves as the sporocarp develops from button stage to maturity (0.11 to 0.062 mg kg^-1^ dw for Pb and 0.013 to 0.006 mg kg^-1^ dw for Sb).

### Relative occurrence of Hg and Se—implication for consumers

Although the relative proportions vary depending on species, the caps of *A. muscaria* generally contain higher concentrations of metallic and metalloid elements (Falandysz et al. 2020) than the stipes except for some elements (Pb Al, Ni, Sr, and uranium). Both Hg and Se occur to a greater proportion in the caps, but while the molar ratio of Hg in cap:stipe remains more or less constant (generally 1.8 ± 0.1), the ratio for Se varies to a greater extent and is highest during stages 2–4 of development. This may relate to a greater demand for seleno-compounds in the caps during these stages of development. The caps of *A. muscaria* has been reported to contain higher concentrations of a number of amino acids such as isoleucine, leucine, valine, alanine, aspartate, asparagine, and threonine, and lipids such as glycerol, choline, and glycerophosphocholine compared to the stipes (Deja et al. 2014).

Although both inorganic and alkylated forms of Hg have been demonstrated to be cytotoxic to neural cells in animals, the potency of the methylated species is far greater (Tong et al. 2016). In exposed humans, MeHg represents around 40% on average, of the THg content of blood (Carneiro et al. 2014) and correlates to a greater extent with adverse effects than inorganic Hg. Although the foods that are most prone to MeHg contamination are those derived from animal and particularly aquatic/marine sources—microorganism-mediated conversion of Hg to methylated forms makes this bioavailable and then bio-concentrated up the marine food web—human exposure will of course depend on individual food preferences and/or availability of different foods. The high consumption rates of fungal species that are foraged by certain populations have been reported before (Zhang et al. 2010), and although edible mushroom species may not contain as high THg levels as fish, the frequency and amounts of consumption may make a significant contribution to an individual’s intake of Hg. The co-occurrence of Se with Hg in the flesh of mushrooms is therefore of considerable significance to consumers, because of the mitigating potential that existing seleno-compounds have on the detrimental health effects of Hg. The large (approximately one order of magnitude) excess of Se relative to Hg observed in *A. muscaria* in this study is therefore of benefit to consumers. However, there are a number of unknowns, when consumed for nutritional purposes; *A. muscaria* will have been processed by boiling in water (which is discarded, in order to eliminate the hallucinogens, muscimol, ibotenic acid, etc.) and possibly further pickled before consumption as seen in some cultures (Phipps et al. 2000).

Mushrooms vary considerably in content of Se and many edible wild species have Se at level below 1 mg kg^-1^ dw, while certain from the family *Boletaceae* are Se-rich with levels exceeding 10 mg kg^-1^ dw (Costa-Silva et al. 2011; Falandysz 2008; Falandysz 2013). The white button mushroom *Agaricus bisporus* (champignon), which is the most popular cultivated edible mushroom and when raw had around 1 to 2 mg of Se per kg^-1^ dw (Costa-Silva et al. 2011; Pankavec et al. 2019), and pickled products had 0.60 mg kg^-1^ dw (median; and medians in the range from 0.19 to 1.6 mg kg^-1^ dw (Pankavec et al. 2019). Thus, eating mushrooms also has value for Se intake to reduce Se deficiency and including inhabitants in Se-depleted zones, while this aspect is worth further research.

These processes (blanching/pickling) are known to leach out both contaminants such as Hg and nutritionally beneficial elements such as potassium, copper, and zinc (Drewnowska et al. 2017a, 2017b). Clearly this would also potentially result in the loss of selenium, although the extent and proportion of this loss relative to Hg are not currently known (as Se in mushrooms is in the form of Se-organic molecules and in a little portion is in insoluble HgSe complexes (Falandysz 2008; Kavčič et al. 2019)). Additionally, a proportion of the THg in *A. muscaria* would occur as MeHg. This proportion is currently known from one study and limited in a number of specimens (n = 3) examined and was as 0.40 to 0.90% (Fischer et al. 1995). However, as a constituent associated with proteins (Charette et al. 2021), MeHg and Se in selenoproteins may not be lost to the same extent as inorganic Hg/Se by aqueous processing such as boiling. These considerations are likely to apply to all edible fungal species.

## Conclusions

The data from this study was derived from *A. muscaria* specimens that were collected in excellent condition during a very short timeframe from the same location. Combined with generally large numbers of individual samples per development stage composite, and low uncertainty of measurement, the data are likely to be representative of the typical concentrations of Hg and Se in the studied development stages of this species. Hg and Se levels were observed to vary during the developmental stages and the variability may relate to the demands in growth. Both Hg and Se levels were lowest during periods of maximum growth of the sporocarp. In common with some other mushroom species, the stipes contained lower concentrations of both elements, which is consistent with the functionality of the stipes as a transport and supporting structure.

Selenium occurs at almost an order of magnitude greater levels than Hg. Due to its role in mitigating the effects of Hg toxicity, this property is of significance to those who consume the species either for nutritional, medicinal, or recreational purposes, although the losses of both these elements during processing are not known.
